# P-1807. Using The Modified Delphi Method to Create Expert Consensus for Candidate Variables for Risk-Adjusted Benchmarking Metrics of Hospital Antimicrobial Consumption

**DOI:** 10.1093/ofid/ofae631.1970

**Published:** 2025-01-29

**Authors:** Michihiko Goto, Tamar F Barlam, Sara E Cosgrove, Heather Davila, Dimitri M Drekonja, Kelly Echevarria, Cassie Cunningham Goedken, Matthew B Goetz, Kevin Hsueh, Kari A Mergenhagen, Lindsay Taylor, David Hernandez, Daniel J Livorsi

**Affiliations:** University of Iowa/Iowa City VAMC, Iowa City, Iowa; Boston Medical Center, Boston, Massachusetts; Johns Hopkins School of Medicine, Baltimore, MD; University of Iowa Carver College of Medicine, Iowa City, Iowa; Minneapolis VA Health Care System, Minneapolis, Minnesota; Pharmacy Benefits Management, Department of Veterans Affairs, San Antonio, Texas; Iowa City VA Medical Center, Iowa City, Iowa; VA Greater Los Angeles Healthcare System, Los Angeles, California; Washington University in St. Louis, Saint Louis, MO; VA WNY Healthcare System, Buffalo, New York; University of Wisconsin School of Medicine and Public Health, Madison, Wisconsin; Iowa City VA Health Care System, Iowa City, Iowa; University of Iowa Carver College of Medicine, Iowa City, Iowa

## Abstract

**Background:**

Studies have shown that both patient- and hospital-level factors can influence the evaluation of antimicrobial consumption, and appropriate risk adjustments are necessary for reliable benchmarking. The curation of candidate variables through experts can be helpful in gaining the trust of users. We conducted a modified two-stage Delphi method to build consensus on candidate variables that should be considered in developing a risk-adjustment model for benchmarking metrics of hospital antimicrobial consumption.

Summary of Expert Consensus for Candidate Variables for Risk-Adjusted Benchmarking Metrics

**Figure:**
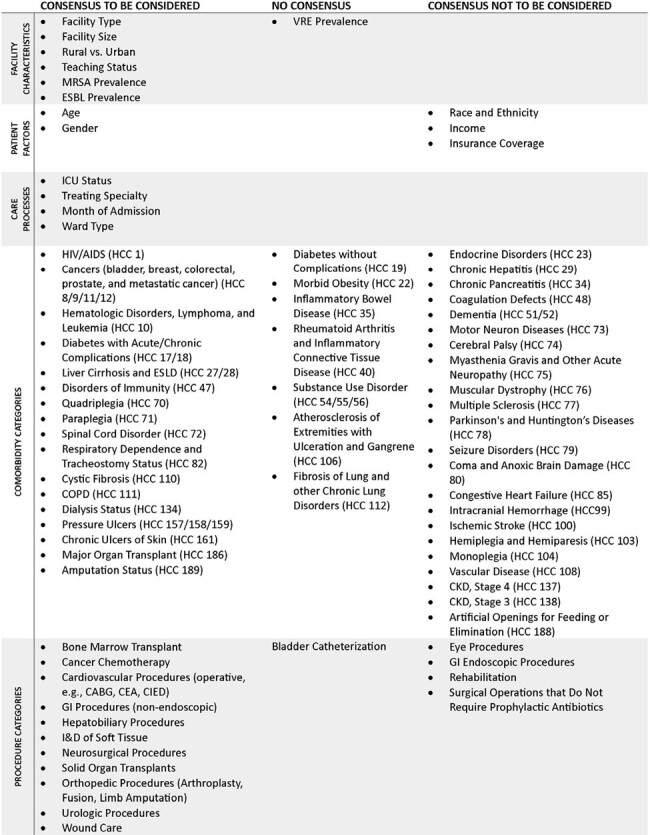

**Methods:**

In preparation, we listed potential variables for consideration from past literature (8 facility characteristics, 6 patient demographics, 4 care processes, 56 comorbidities, 42 procedure categories). We distributed a survey to a 9-member expert panel to rate variables independently to “can be considered” or “should not be included” with comments. Consensus was defined as agreement by 7 or more out of 9 members, and the results with comments were presented to the panel at a conference call for further discussions. The second survey was distributed, focusing on items that did not reach consensus or the panel suggested reconsideration.

**Results:**

At the first survey, 8 facility characteristics, 3 patient demographics, 4 care processes, 27 comorbidities, and 19 procedure categories reached a consensus. The panel discussed the remaining 29 comorbidities and 23 procedure categories and whether any item that reached consensus required reconsideration on a follow-up conference call. In the second survey, 3 facility characteristics, 2 patient demographics, 27 comorbidity categories, and 4 procedure categories were included in the second survey based on the discussion. At the end of the process, 7 facility characteristics, 6 patient demographics, 4 care processes, 49 comorbidities, and 41 procedure categories reached a consensus (Figure).

**Conclusion:**

In this modified two-stage Delphi process, we successfully classified the majority of variables as either to be considered or not to be included in statistical model development. These variables, curated by subject matter experts, can serve as a foundation for further model development for objective evaluation and benchmarking of hospital antimicrobial consumption.

**Disclosures:**

**Michihiko Goto, MD MSCI**, Merck: Grant/Research Support **Daniel J. Livorsi, MD**, Merck: Grant/Research Support

